# Detection of *Mycobacterium ulcerans* in the Environment Predicts Prevalence of Buruli Ulcer in Benin

**DOI:** 10.1371/journal.pntd.0001506

**Published:** 2012-01-31

**Authors:** Heather R. Williamson, Mark E. Benbow, Lindsay P. Campbell, Christian R. Johnson, Ghislain Sopoh, Yves Barogui, Richard W. Merritt, Pamela L. C. Small

**Affiliations:** 1 University of Tennessee, Knoxville, Tennessee, United States of America; 2 University of Dayton, Dayton, Ohio, United States of America; 3 University of Kansas, Lawrence, Kansas, United States of America; 4 Fondation Raoul Follereau, Allada, Benin; 5 Buruli Ulcer Control Programme, Cotonou, Benin; 6 Michigan State University, East Lansing, Michigan, United States of America; University of California San Diego School of Medicine, United States of America

## Abstract

**Background:**

*Mycobacterium ulcerans* is the causative agent of Buruli ulcer (BU). In West Africa there is an association between BU and residence in low-lying rural villages where aquatic sources are plentiful. Infection occurs through unknown environmental exposure; human-to-human infection is rare. Molecular evidence for *M. ulcerans* in environmental samples is well documented, but the association of *M. ulcerans* in the environment with Buruli ulcer has not been studied in West Africa in an area with accurate case data.

**Methodology/Principal Finding:**

Environmental samples were collected from twenty-five villages in three communes of Benin. Sites sampled included 12 BU endemic villages within the Ouheme and Couffo River drainages and 13 villages near the Mono River and along the coast or ridge where BU has never been identified. Triplicate water filtrand samples from major water sources and samples from three dominant aquatic plant species were collected. Detection of *M. ulcerans* was based on quantitative polymerase chain reaction. Results show a significant association between *M. ulcerans* in environmental samples and Buruli ulcer cases in a village (p = 0.0001). A “dose response” was observed in that increasing numbers of *M. ulceran-* positive environmental samples were associated with increasing prevalence of BU cases (R^2^ = 0.586).

**Conclusions/Significance:**

This study provides the first spatial data on the overlap of *M. ulcerans* in the environment and BU cases in Benin where case data are based on active surveillance. The study also provides the first evidence on *M. ulcerans* in well-defined non-endemic sites. Most environmental pathogens are more broadly distributed in the environment than in human populations. The congruence of *M. ulcerans* in the environment and human infection raises the possibility that humans play a role in the ecology of *M. ulcerans*. Methods developed could be useful for identifying new areas where humans may be at high risk for BU.

## Author Summary

Buruli ulcer, a severe, cutaneous disease in West and Central Africa is caused by *Mycobacterium ulcerans*. Person-to-person spread of *M. ulcerans* is rare. There is a strong epidemiological association with residence near slow moving water, but lack of accurate case data in Africa has greatly complicated transmission studies of *M. ulcerans* from the environment to humans. We have combined molecular tools for identification of *M. ulcerans* in the environment with accurate Buruli ulcer case data based on a long standing active surveillance program to map the association between Buruli ulcer and *M. ulcerans* in the environment in Benin. We found a positive association between *M. ulcerans* in the environment and Buruli ulcer cases and show that as the numbers of *M. ulcerans* positive samples/village increase so does the prevalence of Buruli ulcer. Many environmental pathogens are widespread in the environment in the absence of human disease. The failure to obtain definitive proof for *M. ulcerans* in environmental samples where Buruli ulcer is absent raises the intriguing possibility that humans play a role in the distribution of *M. ulcerans*. Sampling methods we have developed could be especially useful for identifying new areas where people may be at risk for Buruli ulcer.

## Introduction

*Mycobacterium ulcerans* is the causative agent of Buruli ulcer, a necrotizing skin disease prevalent in at least 30 subtropical countries [1]. In Africa, close to 30,000 cases were reported between 2005 and 2010 [2]. Cote d'Ivoire, with the highest incidence, reported 2533 cases in 2010. The major virulence determinant in *M. ulcerans* is a macrolide, mycolactone that is responsible for the necrosis and immunosuppression characteristic of Buruli ulcer [3]. Genes for mycolactone biosynthesis form a 110 kb cluster on a large 174KB plasmid [4]. Identification of *M. ulcerans* in the environment is based upon PCR amplification of mycolactone gene sequence, and two insertion sequences (IS*2404* and IS*2606*) present in high copy number in *M. ulcerans* [5]–[8]. Although mycolactone-encoding plasmids have been found in other mycobacterial species in the *M. marinum* complex as well as in unique clades of *M. marinum* none of these species have been identified in Africa.

*M. ulcerans* transmission is still not understood; however it is likely to occur from contact with the environment [1]. There is little evidence of person-to-person transmission though rare cases of possible human-to-human transmission have been described [9]. Residence near an aquatic environment has been identified as a consistent risk factor for infection in Africa [10]–[12]. However, the association of *M. ulcerans* with water is a large-scale (e.g., regional) association; contact with water *per se* has not been directly implicated as a risk factor for Buruli ulcer. In fact, some groups most closely associated with prolonged and frequent water contact such as fisherman, are not at high risk for infection [13], [14].

The completion of the *M. ulcerans* genome sequence in 2004 by Stinear et al. provided a portrait of a species undergoing reductive evolution [15]. The identification of the unique high copy number insertion sequences IS*2404* and IS*2606* in *M. ulcerans* along with genes encoding mycolactone biosynthesis led to the development of molecular tools for identification of *M. ulcerans* in environmental samples [4]. More recently, variable number tandem repeat (VNTR) typing and SNP analysis has been used to discriminate between Ghanaian *M. ulcerans* isolates [6], [16], [17].

In the past 10 years, there have been numerous reports of *M. ulcerans* DNA in aquatic samples collected in Buruli ulcer endemic regions of West Africa [18]–[22]. Using IS2*404*-PCR, *M. ulcerans* DNA has been detected in many species of invertebrates, as well as in fish, snails and frogs [18], [19]. In a collection of 57 hemipterans in a BU endemic area in Benin, Kotlowski et al. detected *M. ulcerans* DNA in 4/5 taxa of predaceous hemipterans [18]. *M. ulcerans* DNA has also been detected in association with water plants, and in a number of aquatic invertebrate species by groups working in Cote d'Ivoire and Cameroon [19]–[21]. More recent standards for identification of *M. ulcerans* DNA in environmental samples require detection of both IS*2404* and sequence associated with enoyl reductase (ER) or ketoreductase (KR) domains from the polyketide synthase genes which encode mycolatone (*mlsA*, *mlsB*). Using these criteria, qPCR was used to detect *M. ulcerans* DNA in one endemic and two non-endemic villages in Ghana [22]. In this study, 148 environmental samples including water (N = 13), detritus (N = 45), tree trunk biofilm (N = 45) and plant biofilm (N = 45) were tested for *M. ulcerans*. *M. ulcerans* was detected in only 1 water sample from an endemic village; all other samples were negative [22].

In the only large-scale study where environmental samples were collected by standard sampling methods, *M. ulcerans* DNA was detected in both BU endemic and non-endemic villages within adjacent districts in Ghana. Although *M. ulcerans* DNA was detected in 12.8% (15/117) of predaceous hemipterans samples, *M. ulcerans* DNA was not detected in 59 of the 89 primarily invertebrate taxa collected [6]. Using conventional PCR, *M. ulcerans* DNA was detected in 8/82 (9.8%) water samples, results comparable to data from the qPCR study reported from Ghana (7.7%) [6], [22]. The most unexpected result from this study was that *M. ulcerans* was detected equally in samples from BU endemic and non-endemic villages. In this study, BU epidemiology was based on passive surveillance. When teams were later sent to the same villages to conduct active case finding, BU cases were detected in nearly all of the villages previously labeled non-endemic.

Although several studies have been published in the past 10 years on detection of *M. ulcerans* DNA in the environment, it is difficult to glean robust, comparative data because of the lack of details on sampling methodology, methods for ecological sampling, lack of data from “control” sites, and lack of accurate epidemiological data.

In the present study we have used standardized, consistent sampling methods, and multiple target, serial qPCR, to identify *M. ulcerans* DNA in environmental samples from 25 villages in Benin. Highly accurate prevalence data, based on the active surveillance program established by the National Buruli Ulcer Program, made it possible to compare the presence of *M. ulcerans* in the environment with Buruli ulcer cases in 22 of these villages.

Environmental samples included water filtrand from major village water sources, and dominant plant samples, along with random invertebrate, excrement, and soil samples. Samples were assayed for *M. ulcerans* DNA and DNA from other mycolactone producing mycobacteria (MPM) using serial, quantitative PCR analysis first targeting IS*2404* followed by the enoyl reductase (ER) domain found on the plasmid responsible for mycolactone. Results of this study show a positive relationship between bacterial distribution among environmental samples and community disease burden. Not only did PCR positive results have high predictive value for BU endemicity, the number of positive samples showed a positive correlation with BU prevalence.

## Materials and Methods

### Site Selection

**Figure 1:**
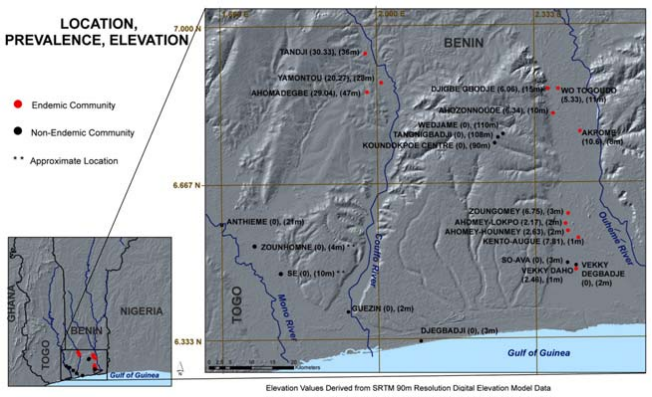
Buruli ulcer prevalence and elevation in Benin where environmental samples were collected April 2009. Prevalence data was available from 22 of the 25 sampled villages.

Environmental samples analyzed in this study were collected from a total of 25 villages. Of the 25 total villages sampled, 22 of these had prevalence (number of Buruli ulcer cases/1000 people) data based on village-based active case surveillance program that had been in place for over five years (Figure 1). In this program, data has been collected monthly in each village on cutaneous lesions with patients being sent to Lalo Health Center for confirmation. Quarterly site visits have been made by health center personnel to validate data collected by community volunteers. Villages were located in the Couffo, Ouhémé, or Mono River basins, near the coast or along a ridge at 100 M adjacent to endemic sites along the Ouhémé River. Quantitative analysis of *M. ulcerans* DNA was performed on samples collected from all villages (N = 25 villages); however, comparisons between *M. ulcerans* presence and abundance and Buruli ulcer prevalence could only be made with 22 of the 25 villages with known prevalence data.

### Elevation Values

Elevation values were derived from 90 m resolution NASA Shuttle Radar Topographic Mission (SRTM) (2000) digital elevation model (DEM) data, acquired from the University of Maryland Global Land cover Facility (http://glcf.umiacs.umd.edu/data/srtm). Elevation sinks were filled before extracting values corresponding to specific village locations using ArcGIS 9.3 software program (ESRI Inc., Redlands, CA).

### Environmental Sampling

#### a. Water filtrand collection

**Figure 2:**
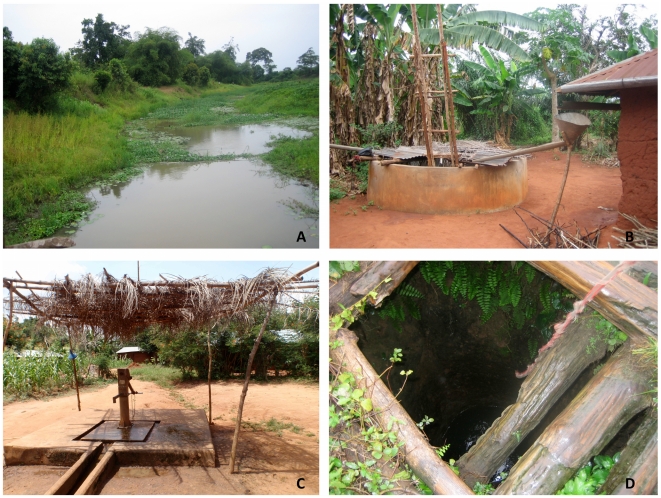
Representative water sources sampled. Water filtrand was collected from rivers/ponds (A), open cisterns (B), water pumps (C), and open wells (D).

For each site, 50 mL of water were passed through a 50 mL syringe fitted with a 1.6 micron filter. This filtrate was then passed through a 0.2 µm filter. Filters were stored in aluminum foil for transport to the University of Tennessee. Water filtrand samples were collected from rivers or ponds (Figure 2a), open cisterns within villages (figure 2b), water pumps (figure 2c), and wells (figure 2d).

#### b. Macrophyte collection

Three to five representatives of the three most dominant plants were collected. Dominant plants were collected from within the edge of the water body or along the bank. Each sample included roots, stems, and leaves when possible. Samples were placed in a plastic, sealable bag and 50 mL sterile water was added. Plant associated biofilm was dislodged from the plants by vigorously rubbing the samples within the bag. Twenty-five milliliters of the resulting liquid suspension were placed into a 50 mL screw cap tube along with a portion of the plant, and preserved in 25 mL, 100% ethanol for analysis by PCR.

#### c. Non-standardized sampling

One to two grams of soil or excrement were collected as well as biofilm from substrates associated with some water sources. Biofilm samples included epilithic communities from wells, well bucket biofilm, and biofilm from barrels used for palm washing. Random, aquatic invertebrate taxa were collected as a composite using a dip net and later sorted and identified in the lab. One small fish and one tadpole were inadvertently trapped and only discovered during sorting. These two vertebrate samples were analyzed for the presence of *M. ulcerans* along with invertebrate samples. No systematic or intentional collection of vertebrates was conducted in this study. IACUC approval is not required for the rare, unintentional collection of vertebrates that may occur during environmental sampling for invertebrates.

### Molecular Analysis

#### a. DNA extraction

Methods for DNA extraction were as described by Williamson et al. [6]. Five hundred microliters of concentrated plant biofilm were used for DNA extraction. Entire portions of water filtrand, soil, invertebrate, vertebrate, biofilm, and excrement samples were used for extraction. *M. ulcerans* Agy*99* and *M. ulcerans* 1615 DNA was extracted as positive controls; and negative controls were included with each extraction.

#### b. Quantitative PCR

Samples were subjected to semi-quantitative PCR using a probe targeting IS*2404* with an internal positive control (IPC) to determine the level of inhibition as previously described [5]. “No template controls” that lacked IS*2404* positive DNA, and “No amplification controls” with IS*2404* DNA and an IPC blocking agent were included.

A positive control was included in quadruplicate with IS*2404* positive DNA and IPC DNA with no blocking agent. DNA of known concentration was also included in duplicate for semi-quantitation.

Samples were loaded in duplicate in a 96 well plate using 3 µL DNA and 22 µL master mix that included 1 µL IPC DNA, 1 µL forward primer, 1 µL reverse primer, 2.5 µL IPC master mix, 2.5 µL *IS*2404 Taqman probe, 1.5 µL water, and 12.5 µL Taqman environmental master mix.

Reaction conditions were such as to detect fluorescence for FAM (IS*2404*), and VIC (IPC) dyes. Semi-quantitative PCR products were detected using an Opticon monitor III (BIORAD) with parameters as follows: 50°C for 2 minutes, 95°C for 10 minutes, and 40 cycles of 95°C for 15 seconds and 60°C for 30 seconds.

Quantitative PCR was carried out on all samples found positive in the IS*2404*-IPC reaction by targeting ER using a Taqman probe with TET dye. A standard curve was generated using serial dilutions of DNA with known copies of ER, created from a purified plasmid template. Standard DNA was run in triplicate. “No template controls” were also included in each run. Forty IS*2404* negative samples were also ran in duplicate in order to determine the specificity of the ER probe.

Loaded wells included 5 µL DNA and 20 uL master mix that included 1 µL forward primer, 1 µL reverse primer, 3 µL water, 2.5 µL Taqman probe, and 12.5 µL Taqman environmental master mix. Quantitative PCR products were detected using Opticon monitor III (BIORAD) with parameters as follows: 95°C for 10 minutes, and 40 cycles of 95°C 15 seconds, 56°C for 30 seconds.

Results were only considered if the standard curve correlation coefficient (R^2^) exceeded or was equal to .99, and if the log linear slope fell within the range of −2.9 and −3.6. DNA in duplicate was rerun in instances where duplicate reactions did not yield similar results, or if above criteria were not met. Extrapolations were made for water filtrand for determination of *M. ulcerans* genome units (GU)/mL, and the remaining samples' quantities were expressed as *M. ulcerans* GU/sample.

A sample was scored positive for *M. ulcerans* DNA if both IS*2404* and ER targets were amplified by PCR.

### Quality Control and Quality Assurance

Standard operating procedures for quality assurance of molecular analyses were strictly followed according to the Quality Assurance/Quality Control Guidance for Laboratories Performing PCR analyses on Environmental Samples and microbial source tracking by the Environmental Protection Agency, USA [23]. Ten-percent of samples were sent to two independent laboratories for evaluation as part of a quality control program (Table S1).

### Statistical Analysis

Statistical analyses were performed using SPSS Statistics 19.0. Chi-square and Bonferroni post-hoc tests were used to determine whether there were significant differences in IS*2404* and ER positivity between sample matrices and matrix positivity and endemicity. The Fisher's exact test was used to determine whether IS*2404* and/or ER positivity was positively associated with Buruli ulcer endemicity. Pearson's correlation was used to determine whether there was a correlation between IS*2404* and ER positivity and Buruli ulcer prevalence, and linear regression was used to model the relationships between IS*2404* and ER with Buruli ulcer prevalence. Significance was defined as p≤0.05.

## Results

### Spatial Distribution of Buruli Ulcer Prevalence and Altitude

Although Buruli ulcer has been consistently associated with residence in low-lying areas where water accumulates, none of the sites previously studied included low-lying swamp areas close to the coast. Altitude was incorporated into our study of 25 villages to determine how broadly the association between low altitude and Buruli ulcer held true. Our results showed a unimodal distribution with respect to altitude. Villages with 5 year BU prevalence less than 15 cases/1000 population were most common either at elevations less than 25 m (Figure 1), or at high elevations (90–100 m). Three villages with BU prevalence greater than 20 cases/1000 population were situated between 20–50 meters.

Three of the non-endemic villages, Athieme, Zounhomne and Se, lie within the Mono River drainage, an area in which Buruli ulcer has never been reported, and another 3 (Wedjame, Tangnigbadji and Koundokpoe Center) are located on a high ridge adjacent to the Oehme River (110 m, 109 m, and 90 m respectively). The remaining 4 non-endemic villages are less than 18 km from the coast and include Guezin in the Couffo delta, Djegbadji on the coast, and Vekky degbadji and So-Ava near the mouth of the Oehme River. Although high BU prevalence is characteristic of communities upstream on the Couffo and Oehme rivers, BU is absent or at very low prevalence in communities near the mouth of these rivers. Water bodies in these communities consist of brackish water most of the year. However, during the rainy season a large influx of fresh water decreases the salinity of these aquatic habitats [24].

### *M. ulcerans* in Environmental Samples Shows Strong Correlation with Endemicity of Buruli Ulcer

Accurate longitudinal case data were available for 22 of the 25 sampled villages (Table 1). From these, 21 villages had analytes that were IS*2404* positive suggesting the possible presence of *M. ulcerans*. IS*2404* positive samples were detected in 9/10 non-endemic villages and 12/12 endemic villages. However, when IS*2404* positive samples were analyzed for the presence of a second target, the enoyl reductase (ER) domain required for mycolactone synthesis, only 2/10 non-endemic villages had samples that were ER positive, whereas 9/12 endemic villages had ER positive samples (Table 1). IS*2404*-PCR showed a positive predictive value of 12/12 (100%) for endemic villages, but IS*2404* alone only accurately predicted 1/10 non-endemic villages (10%). The overall predictive value of IS*2404*-PCR alone for the BU status of all sites was 13/22 (59%). The overall predictive value of ER-PCR on IS*2404* positive samples for BU status was 17/22 (77%; p = 0.0011). The additional use of the ER probe accurately predicted 9/12 endemic sites (75%), and 8/10 (80%) non-endemic villages (p = 0.0574).

**Table 1:** Relationship of IS*2404* and ER-PCR results for *Mycobacterium ulcerans* and Buruli ulcer endemicity per village.

Numerical Village Designation	Village Name	Buruli ulcer Prevalence (cases/1000 pop)	#IS*2404* positive/total sampled (%)	#ER positive/IS*2404* positive (% pos)
1	So Ava	0	4/22 (18)	0/4 (0)
2	Vekky Degbadje	0	2/10 (20)	0/2 (0)
3	Tangnigbadji	0	1/9 (11)	0/1 (0)
4	Koundokpoe Center	0	2/11 (18)	1/2 (50)
5	Wedjame	0	0/8 (0)	0/0 (0)
6	Athieme	0	3/9 (33)	0/3 (0)
7	Zounhomne	0	7/8 (88)	1/7 (14)
8	Se	0	2/8 (25)	0/2 (0)
9	Guezin	0	4/8 (50)	0/4 (0)
10	Djebadji	0	5/8 (63)	0/5 (0)
11	Ahomey Lokpo	2.17	1/8 (13)	1/1 (100)
12	Vekky Daho	2.46	4/7 (57)	1/4 (25)
13	Ahomey-Hounmey	2.63	11/16 (69)	0/11 (0)
14	WoTogoudo	5.33	2/14 (14)	0/2 (0)
15	Djigbe Gbodje	6.06	2/10 (20)	2/2 (100)
16	Ahozonnoude	6.34	4/14 (29)	1/4 (25)
17	Zoungomey	6.75	7/13 (54)	1/7 (14)
18	Kento Augue	7.81	5/9 (56)	0/5 (0)
19	Akpome	10.6	8/16 (50)	6/8 (75)
20	Yamounto	20.27	12/15 (80)	6/12 (50)
21	Tchi-Ahomadegbe	29.04	5/10 (50)	2/5 (40)
22	Tandji	30.33	14/26 (54)	10/14 (71)

PCR was first used targeting IS*2404*. Those found to be IS*2404* positive were further assayed using PCR targeting the enoyl reductase domain involved in mycolactone synthesis. Results represent the number positive from the total number assayed in a particular village.

The average ct values of IS*2404* positive samples was 37.25 suggesting the possibility that lower bacterial abundance may explain the failure to detect ER from some IS*2404* positive samples, rather than a lack of specificity to *M. ulcerans* or other MPMs. There was no significant difference, however, between the average ct values in samples collected from endemic or non-endemic habitats (p = 0.08).

It was possible to estimate the numbers of *M. ulcerans* DNA in environmental samples using ER-PCR in 92% (12/13) of IS*2404* positive samples whose ct values ranged from 27.68 to 34.85 (Table S2). The ability to estimate bacterial burden fell as the ct value increased. Bacterial numbers could be estimated by ER-PCR in 44% (11/25) of samples whose IS*2404* ct values ranged from 35.43 and 36.97 and only in 19% (15/78) of samples whose IS*2404* ct values ranged from 37.03 and 39.88 (Table S2). Forty IS*2404* negative samples were tested with ER-PCR and none were found positive.

### Distribution of *M. ulcerans* Predicts Level of Buruli Ulcer Prevalence

**Figure 3:**
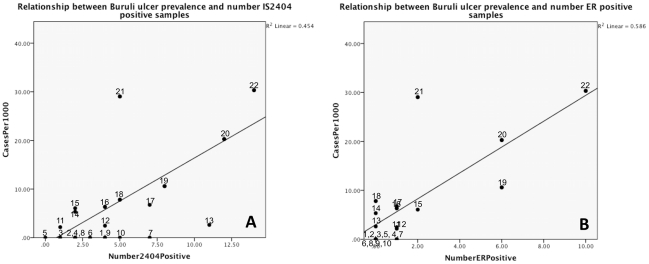
Linear relationship between Buruli ulcer prevalence and number of IS*2404* and ER positive samples. (A) Coefficient of Determination (R^2^) IS*2404* = 0.454; F = 21.652; df = 1; p = 0.0001. (B) Coefficient of Determination (R^2^) ER = 0.586; F = 36.748; df = 1; and p = 0.0001. Numbers correspond to village designation shown in Table 1.

If *M. ulcerans* were contracted through environmental exposure, it would be expected that the extent of *M. ulcerans* in the environment would correlate with the extent of Buruli ulcer disease in humans if surveillance and reporting were accurate. To test this hypothesis we compared PCR positivity with BU prevalence in that site (Table 1). The numbers of samples taken per site differed, because sites differed in the number of water sources. However, as seen in Figure 3, there was a reasonable and statistically significant linear relationship between numbers of *M. ulcerans* positive samples and the prevalence of Buruli ulcer cases. *M. ulcerans* DNA was found in 40–75% of the samples tested in four highly endemic communities (Akpome, Yamanto, Tchi-Ahomadegbe, and Tandji) with BU prevalence above 10/1000. With few exceptions, less than 25% of environmental samples were positive from sites with BU prevalence below 10/1000 (Table 1). Using Pearson's test of correlation, IS*2404* positivity was strongly correlated with Buruli ulcer prevalence (ρ = 0.674; p = 0.0001) as was ER positivity (ρ = 0.765; p = 0.0001). There was a significant linear relationship of Buruli ulcer prevalence and IS*2404* and ER positivity, with 45% and 59% of the variation in BU prevalence explained by IS*2404* (R^2^ = 0.454) and ER positivity (R^2^ = 0.586), respectively. Thus, although the numbers of IS*2404* positive samples/site were correlated with BU prevalence, serial PCR using IS*2404*-PCR followed by ER-PCR on IS*2404* positive samples substantially improved the ability (by 14%) to predict Buruli ulcer prevalence at a site based on PCR results from environmental samples (Figure 3).

### Analysis of Water Filtrand Is a Robust Method for Detection of *M. Ulcerans* in the Environment

In order to determine the presence and abundance of *M. ulcerans* within and around water sources, 275 samples were collected from 25 villages (Table 2). Samples were collected from eight different matrices. Water filtrand samples had a consistently higher positivity than any other matrix assayed. Twenty of the forty-seven well water filtrand samples collected were positive for IS*2404*. From these 20, 14 were positive for ER DNA, and the mean bacterial load was 1.68×10^3^ GU/mL. Thirty-five pond or river filtrand samples were positive for IS*2404*, and 15 of these were also positive for ER. Mean genome units were 1.68×10^3^ GU/mL. Nine water filtrand samples were collected from cisterns. Of these, four contained IS*2404*, and one contained both IS*2404* and ER DNA (Table 2).

**Table 2:** Environmental samples containing mycolactone-producing mycobacteria DNA from all samples per matrix.

Matrix	# IS*2404* pos/total sampled (%)	# ER Pos/total IS*2404* pos (%)	Mean GU Quantity (Range)
**Water filtrand**	**59/130 (45.4)**	**30/59 (51)**	1.64×10^3^GU/mL (1.01×10^2^–1.33×10^4^)
Well filtrand	20/47 (42.6)	14/20 (70)	1.68×10^3^GU/mL (1.25×10^2^–1.3×10^4^)
Pond/River Filtrand	35/74 (47.3)	15/35 (43)	1.68×10^3^GU/mL (1.01×10^2^–1.14×10^4^)
Cistern filtrand	4/9 (44.4)	1/4 (25)	439.04* GU/mL
**Biofilm**	6/19 (31.6)	3/6 (50)	4.04×10^4^ GU/sample (2.20×10^4^–7.7×10^4^)
**Soil**	12/46 (26.1)	3/12 (25)	3.18×10^6^GU/sample (4.0×10^4^–9.4×10^6^)
**Macrophytes**	36/69 (52.2)	2/36 (5.6)	1.07×10^4^ GU/sample (1.07×10^4^–1.08×10^4^)
**Excrement**	1/2 (50)	0/1 (0)	NA
**Invertebrate/Vertebrate**	1/9 (11.1)	0/1 (0)	NA

Mean quantities of MPM DNA found in each positive sample are included.

NA: not applicable.

*Values are measured as Genome units (GUs)/sample. Value derived from only one sample positive.

Three of the six IS*2404* positive biofilm samples (N = 19 total collected) contained ER DNA with a mean quantity of 4.04×10^4^ GU/sample. Twelve of the 46 soil samples were IS*2404* positive. Out of these, three were also positive for ER. Soil samples contained the highest quantity of *M. ulcerans* DNA with a mean quantity of 3.18×10^6^ GU/sample. Thirty-six of 69 macrophytes contained IS*2404* DNA. Of these, two were also positive for ER. One macrophyte sample had 1.07×10^4^ GU/sample and one had 1.08×10^4^ GU/mL, with a mean quantity of 1.07×10^4^ GU/sample. One of two excrement samples and one of nine invertebrate/vertebrate samples were positive for IS*2404*, however neither matrix was positive for ER. Collectively, water filtrand had the highest positivity from all other matrices sampled, and well filtrand had the highest overall positivity. There was no significant difference in IS*2404* positivity between matrices (p = 0.071), but there were significantly more ER positive samples from well filtrand than from soil and macrophytes (p = 0.004 and .0001 respectively), and pond/river filtrand had significantly more ER positive samples than macrophytes (p = 0.001). Matrices were also analyzed with respect to positivity and endemicity. There was a significantly higher number of positive samples from well filtrand collected in endemic villages compared to well filtrand samples analyzed from non-endemic villages (p = 0.001). Neither IS*2404* nor ER positivity differed significantly between endemic and non-endemic sites for any other matrix.

## Discussion

This is the first large-scale spatial study in West Africa in which the distribution of *M. ulcerans* in the environment and cases of Buruli ulcer were mapped using longitudinal Buruli ulcer case data based on active surveillance. A major finding from this study was the identification of a positive relationship between the presence and abundance of *M. ulcerans* DNA in a village, and the numbers of Buruli ulcer cases in humans. These results contrast significantly with those of our earlier large-scale study conducted in Ghana [6], [25] where *M. ulcerans* DNA was detected in the environment equally in endemic and non-endemic villages. How can these discrepancies be explained? We think the primary reason for these different findings lies in the methods used to detect and report BU cases in Benin and Ghana. In Benin, a program of monthly active case detection using community volunteers has been well established since 2004. Active surveillance has generated highly accurate case data though it is labor intensive. In Ghana, BU cases are spread over a much larger geographic region, and case detection has relied on passive surveillance, a much less accurate epidemiological method [6], [26]–[28].

There were also differences between the environmental sampling conducted in Benin and Ghana. Although water filtrand, plants and soil were sampled using similar methodology in both countries, invertebrates made up a large portion of the samples collected in Ghana whereas standardized sampling of invertebrates was not conducted in Benin [6]. The results from the study in Ghana were based on conventional PCR whereas qPCR was used for sample analysis in Benin. Of these factors we consider the difference in accuracy of case detection to be the most likely explanation for the fact that a significant correlation between BU cases and the presence of *M. ulcerans* in the environment was found in Benin but not in Ghana. More recently, our team, as well as other Ghanaian field teams, has discovered Buruli ulcer in many Ghanaian communities previously designated non-endemic. Results from this study provide strong advocacy for the use of prevalence data from active case surveillance as a basis for any study attempting to link Buruli ulcer with *M. ulcerans* in environmental samples.

Geography may play a role in the distribution of *M. ulcerans* as well as in the distribution of Buruli ulcer prevalence. Villages with less than 15/1000 BU cases were located at elevations less than 25 m or at elevations greater than 80 meters, and a similar distribution was found for *M. ulcerans*. Villages located at the lowest elevations were, in general, close to the coast where the presence of high salinity could be inhibitory to the growth of *M. ulcerans*, or to the presence of *M. ulcerans* associated habitats. BU has been extremely rare in people living on the coast in West Africa. Our results differed from a study by Sopoh et al. where the prevalence of Buruli ulcer was correlated with lowland areas at an altitude less than 50 m [29]. However, the apparent discrepancies between these studies may lie in the difference in scope. In the Sopoh paper, study sites were located within a more narrow geographic area compared to the sites presented in this paper, and none of those sites described by Sopoh et al. were located within 30 km of the coast. Ten of the sites in this study were below 8 m and four were at sea level. None of the sites in the Sopoh et al. study were located at such low elevations. Additionally, elevation values derived from SRTM data were less precise than values obtained from the Trimble GPS unit employed by Sopoh et al. Therefore, SRTM error may have also contributed to differences in the study outcomes. Despite this limitation, our results were sufficient to confirm data showing the low prevalence of Buruli ulcer in coastal communities in West Africa.

Our study confirms the necessity of serial testing with multiple PCR probes when evaluating environmental samples for presence of an organism [5]–[7], [22]. Although IS*2404* positive samples were detected in nine of ten aquatic habitats located in non-endemic villages, further evidence for *M. ulcerans* in these samples could only be obtained in two samples using ER-PCR. The copy number of IS*2404* is at least 18 fold higher than that of the ER sequence. Threshold values (ct) for some IS*2404* positive samples were high, suggesting the presence of too few organisms for ER detection. IS*2404* has been found in several mycobacterial groups closely related to *M. ulcerans* in the *M. marinum* complex associated with aquatic environments. However a second explanation for the presence of IS*2404* positive/ER negative samples in non-endemic areas is that they may reflect the presence of mycobacterial species in the *M. marinum* complex which are closely related to *M. ulcerans* but do not cause Buruli ulcer [6].

This is the first report of *M. ulcerans* DNA in water filtrand from wells and cisterns. However, *M. ulcerans* DNA has been associated with surface waters in several studies [6], [7], [22]. Ground and surface water exchange has been well documented [30]–[33] and this exchange is defined by floodplain geomorphology [34]. It is likely that the presence *of M. ulcerans* in these water sources is related to fluctuations in hydroperiod that lead to exchange of *M. ulcerans* or *M. ulcerans* DNA within the surface-groundwater continuum. Digging of wells, pits, mineral mining, or groundwater detraction converts groundwater to surface water, thus bringing the communities within groundwater to the surface, and affecting the biodiversity within the aquatic habitat [34].

Although results in this paper are consistent with results from many other studies reporting *M. ulcerans* DNA in natural water sources and water filtrand [6]–[8], our data do not support a role for transmission of *M. ulcerans* through direct contact with water or suggest that *M. ulcerans* grows freely in water. Genome units of *M. ulcerans* in water were between 100–1000/ml. In contrast aquatic pathogens such as *Vibrio cholera* are often present in numbers greater than 10^6^/ml [35]. Our results are more consistent with the hypothesis that organisms detected in water are swept into aquatic sites through run-off from precipitation or sloughing from biofilms within the aquatic habitat. Nonetheless, the fact that water run-off collects organisms from a considerable area provides a simple method for initial screening of a site to determine the likelihood of *M. ulcerans* in a community.

One of the most intriguing aspects of our study is the clue it provides to understanding the relationship between *M. ulcerans* in the environment and *M. ulcerans* in humans. A primary feature of many environmental pathogens such as *Clostridium tetani*, *Francisella tularensis*, or *Borrelia burgdorferi*, is that human infection represents a dead end host for the pathogen [36]–[38]. Consistent with this relationship, the pathogen is often detected in the environment in the absence of human infection. However, not only did we find a strong relationship between the presence of *M. ulcerans* in the environment and the presence of Buruli ulcer in humans, we also found that the detection of *M. ulcerans* DNA in multiple sample types within a single village was a strong predictor of high Buruli ulcer case burden. This finding raises the interesting possibility that humans play an active role in the distribution of *M. ulcerans* in the environment. In recent studies, we have identified very high levels of *M. ulcerans* in environmental sites characterized as “high human activity” areas (unpublished data). This observation is consistent with the widely reported association between Buruli ulcer and anthropomorphic changes in the environment such as sand winnowing, gold mining [39], [40], rice agriculture [41] or other landscape disturbance [42], [43] and suggests that the relationship between *M. ulcerans* and the environment and *M. ulcerans* in humans may be more complex than previously appreciated.

## Supporting Information

Table S1 **Average qPCR threshold cycle (Ct) values from samples analyzed for quality control.** Two additional, independent laboratories analyzed samples. Quantitative PCR was performed targeting the enoyl reductase domain (ER). ^1^Pamela Small Laboratory, University of Tennessee; ^2^Todd Reynolds Laboratory, University of Tennessee; ^3^University of Tennessee Genomics Hub; ND: not detected; NA: not analyzed.
(DOC)

Table S2 **Threshold cycle (Ct) values when the level of fluorescence first began to significantly increase.** Ct results from qPCR using probes for IS*2404* and internal positive control (IPC) for detection of inhibition as well as a probe targeting the enoyl reductase (ER) domain are shown. Abundance of genome units per sample or mL of ER was also included when applicable. Only samples with *IS2404* ct values above zero are shown.
(DOC)
