# Supplementary material for: Detection of Mycobacterium ulcerans in the Environment Predicts Prevalence of Buruli Ulcer in Benin
Source: PLoS Negl Trop Dis. 2012 Jan 31;6(1):e1506. doi: 10.1371/journal.pntd.0001506 (PMC3269429; doi:10.1371/journal.pntd.0001506)
Supplement: Table S1 — Average qPCR threshold cycle (Ct) values from samples analyzed for quality control. Two additional, independent laboratories analyzed samples. Quantitative PCR was performed targeting the enoyl reductase domain (ER). 1Pamela Small Laboratory, University of Tennessee; 2Todd Reynolds Laboratory, University of Tennessee; 3University of Tennessee Genomics Hub; ND: not detected; NA: not analyzed. (DOC) [file pntd.0001506.s001.doc]

**Supplemental Table 1.** **Average qPCR threshold cycle (Ct) values from samples analyzed for quality control.**

| **Community NDme** | **Sample Description** | **PS Lab^1^ Ave ER Ct** | **TR Lab^2^ Ave ER Ct** | **UTGH Lab^3^ Ave ER Ct** |
| --- | --- | --- | --- | --- |
| So Ava | Soil | ND | ND | ND |
| Vekky Degbadje | Crab | ND | ND | ND |
| Ahomey-Hounmey | Unidentified macrophyte | ND | ND | ND |
| Ahomey Hounmey | Pond Filtrand | ND | ND | ND |
| Ahomey Lokpo | Excrement | ND | ND | ND |
| Zoungomey | *Eichornia* sp. | ND | ND | ND |
| Tagnigbadji | Well Filtrand | ND | ND | ND |
| Koundokpoe Center | Cistern Filtrand | ND | ND | ND |
| Wedjame | Well Filtrand | ND | ND | ND |
| Wedjame | Cistern Filtrand | ND | ND | ND |
| Akpome | River Filtrand | 35.05 | 35.60 | 35.80 |
| Akpome | Well Filtrand | 34.95 | 35.97 | 36.85 |
| Akpome | Clay | ND | ND | ND |
| Ahozonnoude | River Filtrand | ND | ND | ND |
| Djigbe Gbodje | Well Filtrand | ND | ND | ND |
| Agoundji | River Filtrand | 35.00 | 35.64 | 34.70 |
| Agbata | Cistern Filtrand | ND | ND | ND |
| Tchi  Ahomadegbe | River Filtrand | ND | ND | ND |
| Tchi  Ahomadegbe | Soil | ND | ND | ND |
| Yamounto | Well Filtrand | 33.32 | 31.42 | NA |
| Yamounto | River Filtrand | 33.50 | 33.72 | 34.66 |
| Tanji | Well Filtrand | 35.81 | 34.79 | 37.12 |
| Tanji | Clay | 34.87 | 34.45 | 34.26 |
| Zounhomne | Soil | ND | ND | ND |
| Zounhomne | *Nymphaea* sp. | ND | ND | ND |
| Guezin | River Filtrand | ND | ND | ND |
| Guezin | Soil | ND | ND | ND |

Samples were analyzed by two additional, independent laboratories. Quantitative PCR was performed targeting the enoyl reductase domain (ER). ^1^Pamela Small Laboratory, University of Tennessee; ^2^Todd Reynolds Laboratory, University of Tennessee; ^3^University of Tennessee Genomics Hub; ND: not detected; NA: not analyzed.
