# Supplementary material for: Detection of Mycobacterium ulcerans in the Environment Predicts Prevalence of Buruli Ulcer in Benin
Source: PLoS Negl Trop Dis. 2012 Jan 31;6(1):e1506. doi: 10.1371/journal.pntd.0001506 (PMC3269429; doi:10.1371/journal.pntd.0001506)
Supplement: Table S2 — Threshold cycle (Ct) values when the level of fluorescence first began to significantly increase. Ct results from qPCR using probes for IS2404 and internal positive control (IPC) for detection of inhibition as well as a probe targeting the enoyl reductase (ER) domain are shown. Abundance of genome units per sample or mL of ER was also included when applicable. Only samples with IS2404 ct values above zero are shown. (DOC) [file pntd.0001506.s002.doc]

| **Community Name** | **Sample Name** | **IPC Ct** | **IS*2404* Ct** | **Ave. ER Ct** | **Ave. GU/sample** | **Ave.**  **GU/ML** |
| --- | --- | --- | --- | --- | --- | --- |
| So Ava | grass | 24.33 | 39.07 | NA | NA | NA |
| So Ava | grass | 23.83 | 39.08 | NA | NA | NA |
| So Ava | excrement | 24.53 | 39.45 | NA | NA | NA |
| So Ava | unidentified macrophyte | 24.78 | 39.59 | NA | NA | NA |
| Vekky Daho | river filtrand | 24.53 | 37.35 | NA | NA | NA |
| Vekky Daho | *Lemna* sp. | 24.75 | 38.76 | 37.98 | 1.08E+04 | NA |
| Vekky Daho | grass | 24.41 | 39.23 | NA | NA | NA |
| Vekky Daho | grass | 24.27 | 36.46 | NA | NA | NA |
| Vekky Degbadje | well filtrand | 24.43 | 38.76 | NA | NA | NA |
| Vekky Degbadje | crab | 23.93 | 37.03 | NA | NA | NA |
| Ahomey-Hounmey | pond filtrand | 23.12 | 38.01 | NA | NA | NA |
| Ahomey-Hounmey | grass | 23.95 | 37.52 | NA | NA | NA |
| Ahomey-Hounmey | *Pistia* sp | 23.98 | 38.37 | NA | NA | NA |
| Ahomey-Hounmey | pond filtrand | 23.59 | 38.02 | NA | NA | NA |
| Ahomey-Hounmey | pond filtrand | 23.66 | 39.16 | NA | NA | NA |
| Ahomey-Hounmey | unidentified macrophyte | 23.69 | 37.14 | NA | NA | NA |
| Ahomey-Hounmey | unidentified macrophyte | 23.91 | 38.73 | NA | NA | NA |
| Ahomey-Hounmey | *Lemna* sp. | 22.87 | 38.48 | NA | NA | NA |
| Ahomey-Hounmey | soil | 23.99 | 37.75 | NA | NA | NA |
| Ahomey-Hounmey | river filtrand | 23.84 | 39.58 | NA | NA | NA |
| Ahomey-Hounmey | river filtrand | 23.37 | 30.32 | NA | NA | NA |
| Ahomey Lokpo | soil dip | 23.59 | 39.00 | 36.66 | 3.97E+04 | NA |
| Zoungomey | river filtrand | 23.76 | 38.97 | NA | NA | NA |
| Zoungomey | well filtrand | 23.24 | 37.89 | NA | NA | NA |
| Zoungomey | well filtrand | 23.57 | 36.14 | 39.1 | 6.30E+03 | 1.26E+02 |

**Supplemental Table 2.** **Threshold cycle (Ct) values when the level of fluorescence first began to significantly increase.**

| **Community Name** | **Sample Name** | **IPC Ct** | **IS*2404* Ct** | | **Ave. ER Ct** | | **Ave.**  **GU/sample** | | **Ave.**  **GU/ML** |
| --- | --- | --- | --- | --- | --- | --- | --- | --- | --- |
| Zoungomey | *Eichhornia* sp. | 23.74 | 37.54 | NA | | NA | | NA | |
| Zoungomey | *Eichhornia* sp. | 23.5 | 39.29 | NA | | NA | | NA | |
| Zoungomey | soil | 24.01 | 37.99 | NA | | NA | | NA | |
| Zoungomey | swab | 22.74 | 37.31 | NA | | NA | | NA | |
| Kento Augue | river filtrand | 22.44 | 39 | NA | | NA | | NA | |
| Kento Augue | unidentified macrophyte | 23.28 | 37.84 | NA | | NA | | NA | |
| Kento Augue | unidentified macrophyte | 23.2 | 38.89 | NA | | NA | | NA | |
| Kento Augue | unidentified macrophyte | 23.27 | 38.33 | NA | | NA | | NA | |
| Kento Augue | soil | 23.4 | 38.59 | NA | | NA | | NA | |
| Tangnigbadji | well filtrand | 22.95 | 36.64 | NA | | NA | | NA | |
| Koundokpoe cntr | well filtrand | 24.15 | 39.8 | NA | | NA | | NA | |
| Koundokpoe cntr | cistern filtrand | 23.82 | 39.06 | 36.87 | | 2.20E+04 | | 4.39E+02 | |
| Akpome | river filtrand | 23.7 | 34.85 | 35.99 | | 6.67E+04 | | 1.33E+03 | |
| Akpome | river filtrand | 23.39 | 34.69 | 35.05 | | 1.03E+05 | | 2.07E+03 | |
| Akpome | river filtrand | 23.88 | 35.49 | NA | | NA | | NA | |
| Akpome | well filtrand | 23.14 | 37.96 | 38.48 | | 1.00E+04 | | 2.01E+02 | |
| Akpome | well filtrand | 23.6 | 33.99 | 34.95 | | 1.12E+05 | | 2.23E+03 | |
| Akpome | river filtrand | 23.8 | 35.79 | 37.43 | | 1.96E+04 | | 3.93E+02 | |
| Akpome | river filtrand | 23.35 | 37.82 | 39.27 | | 5.28E+03 | | 1.06E+02 | |
| Akpome | river filtrand | 23.71 | 37.3 | NA | | NA | | NA | |
| Ahozonnoude | river filtrand | 23.58 | 38.86 | NA | | NA | | NA | |
| Ahozonnoude | well filtrand | 23.31 | 39.53 | NA | | NA | | NA | |
| Ahozonnoude | well biofilm | 23.74 | 37.31 | NA | | NA | | NA | |
| Ahozonnoude | biofilm | 23.29 | 38.52 | 37.45 | | 2.27E+04 | | NA | |
| Mesabo | river filtrand | 23.97 | 38.22 | 37.62 | | 1.68E+04 | | 3.36E+02 | |
| Mesabo | river filtrand | 23.83 | 36.72 | 36.51 | | 3.86E+04 | | 7.72E+02 | |
| Mesabo | soil | 23.17 | 27.68 | 28.66 | | 9.36E+06 | | NA | |

| **Community Name** | **Sample Name** | **IPC Ct** | **IS*2404* Ct** | **Ave. ER Ct** | **Ave. GU/sample** | **Ave.**  **GU/ML** |
| --- | --- | --- | --- | --- | --- | --- |
| Djigbe Gbodje | biofilm | 23.55 | 39.81 | 37.82 | 2.18E+04 | NA |
| Djigbe Gbodje | roots | 22.34 | 36.72 | 38.5 | 1.07E+04 | NA |
| Agoundji | river filtrand | 22.6 | 33.71 | 35 | 1.22E+05 | 2.44E+03 |
| Agoundji | river filtrand | 23.04 | 36.97 | 36.45 | 4.49E+04 | 8.98E+02 |
| Agoundji | river filtrand | 21.44 | 35.88 | 35.79 | 7.00E+04 | 1.40E+03 |
| WoTogoudo | river filtrand | 22.27 | 38.94 | NA | NA | NA |
| WoTogoudo | polygonum | 22.49 | 38.92 | NA | NA | NA |
| Agbata | cistern filtrand | 24.11 | 38.43 | NA | NA | NA |
| Agbata | cistern filtrand | 23.17 | 35.69 | NA | NA | NA |
| Agbata | cistern filtrand | 23.71 | 38.14 | NA | NA | NA |
| Agbata | biofilm | 24.6 | 38.03 | NA | NA | NA |
| Agbata | soil | 24.49 | 36.92 | NA | NA | NA |
| Tchi Ahomadegbe | well filtrand | 24.67 | 35.76 | 38.6 | 1.53E+04 | 3.07E+02 |
| Tchi Ahomadegbe | well filtrand | 24.19 | 32.95 | 36.54 | 7.06E+04 | 1.41E+03 |
| Tchi Ahomadegbe | pond filtrand | 24.48 | 36.51 | NA | NA | NA |
| Tchi Ahomadegbe | *Nymphaea* sp. | 24.45 | 38.42 | NA | NA | NA |
| Tchi Ahomadegbe | *Commelina* sp. | 24.19 | 38.31 | NA | NA | NA |
| Yamounto | well filtrand | 24.45 | 31.2 | 33.32 | 6.64E+05 | 1.33E+04 |
| Yamounto | well filtrand | 24.52 | 33.68 | 38.01 | 2.29E+04 | 4.58E+02 |
| Yamounto | well filtrand | 24.16 | 37.68 | 36.7 | 5.70E+04 | 1.14E+03 |
| Yamounto | river filtrand | 24.21 | 31.17 | 33.5 | 5.72E+05 | 1.14E+04 |
| Yamounto | river filtrand | 23.72 | 31.14 | 35.46 | 1.36E+05 | 2.72E+03 |
| Yamounto | river filtrand | 24.18 | 38.86 | NA | NA | NA |
| Yamounto | unidentified macrophyte | 24.37 | 38.11 | NA | NA | NA |
| Yamounto | palm wash tub biofilm | 24.08 | 32.37 | 36.29 | 7.67E+04 | NA |
| Yamounto | *Pistia* sp. | 24.61 | 36.57 | NA | NA | NA |

| **Community Name** | **Sample Name** | **IPC Ct** | **IS*2404* Ct** | **Ave. ER Ct** | **Ave. GU/sample** | **Ave.**  **GU/ML** |
| --- | --- | --- | --- | --- | --- | --- |
| Yamounto | *Polygonum* sp. | 24.37 | 38.18 | NA | NA | NA |
| Yamounto | grass | 24.63 | 35.88 | NA | NA | NA |
| Yamounto | soil | 25.06 | 37.74 | NA | NA | NA |
| Tandji | well filtrand | 23.81 | 36.11 | 35.81 | 6.97E+04 | 1.39E+03 |
| Tandji | well filtrand | 21.43 | 36.27 | 36.63 | 3.89E+04 | 7.79E+02 |
| Tandji | well filtrand | 23.32 | 37.31 | 39.29 | 6.68E+03 | 1.34E+02 |
| Tandji | well filtrand | 23.03 | 35.74 | 36.99 | 3.07E+04 | 6.15E+02 |
| Tandji | well filtrand | 23.42 | 37.03 | NA | NA | NA |
| Tandji | well filtrand | 23.7 | 37.59 | 38.6 | 1.17E+04 | 2.34E+02 |
| Tandji | well filtrand | 22.27 | 37.72 | 35.99 | 6.12E+04 | 1.22E+03 |
| Tandji | well biofilm | 23.31 | 39.49 | NA | NA | NA |
| Tandji | river filtrand | 23.74 | 39.47 | 38.86 | 9.49E+03 | 1.90E+02 |
| Tandji | river filtrand | 23.13 | 39.17 | 36.92 | 3.19E+04 | 6.37E+02 |
| Tandji | river filtrand | 23.4 | 38.1 | 38.86 | 8.31E+03 | 1.66E+02 |
| Tandji | rice stalks | 23.05 | 38.62 | NA | NA | NA |
| Tandji | clay | 23.57 | 35.99 | 34.87 | 1.33E+05 | NA |
| Tandji | plant on rice bund | 23.21 | 38.59 | NA | NA | NA |
| Athieme | unidentified macrophyte | 22.93 | 39.88 | NA | NA | NA |
| Athieme | unidentified macrophyte | 22.93 | 38.6 | NA | NA | NA |
| Athieme | soil | 23.22 | 37.47 | NA | NA | NA |
| Zounhomne | river filtrand | 23.56 | 36.09 | NA | NA | NA |
| Zounhomne | river filtrand | 23.7 | 33.55 | 37.69 | 1.42E+04 | 2.85E+02 |
| Zounhomne | river filtrand | 23.72 | 35.43 | NA | NA | NA |
| Zounhomne | *Eichhornia* sp. | 23.63 | 37.27 | NA | NA | NA |
| Zounhomne | *Nymphaea* sp. | 24.65 | 39.71 | NA | NA | NA |
| Zounhomne | soil | 23.86 | 38.68 | NA | NA | NA |
| Zounhomne | *Polygonum* sp. | 23.56 | 36.79 | NA | NA | NA |
| Se | *Ipomea* sp. | 23.37 | 38.61 | NA | NA | NA |
| Se | *Ipomea* sp. | 23.67 | 38.75 | NA | NA | NA |
| Guezin | river filtrand | 23.32 | 36.83 | NA | NA | NA |
| Guezin | river filtrand | 23.74 | 39.3 | NA | NA | NA |
| Guezin | grass | 23.22 | 37.93 | NA | NA | NA |

| **Community Name** | **Sample Name** | **IPC Ct** | **IS*2404* Ct** | **Mean ER Ct** | **Mean GU/sample** | **Mean**  **GU/ML** |
| --- | --- | --- | --- | --- | --- | --- |
| Guezin | grass | 23.48 | 37.62 | NA | NA | NA |
| Djebadji | river filtrand | 23.66 | 37.82 | NA | NA | NA |
| Djebadji | river filtrand | 23.82 | 36.78 | NA | NA | NA |
| Djebadji | grass | 23.46 | 37.72 | NA | NA | NA |
| Djebadji | soil | 23.64 | 36.64 | NA | NA | NA |
| Djebadji | soil | 23.65 | 38.78 | NA | NA | NA |

Ct results from qPCR using probes for IS*2404* and internal positive control (IPC) for detection of inhibition as well as a probe targeting the enoyl reductase (ER) domain are shown. Abundance of genome units per sample or mL of ER was also included when applicable. Only samples with *IS2404* ct values above zero are shown.
